# Evaluating the Primary Prevention of Ischemic Stroke of Oral Antithrombotic Therapy in Head and Neck Cancer Patients with Radiation Therapy

**DOI:** 10.1155/2016/6205158

**Published:** 2016-11-21

**Authors:** Chin-Wei Hsu, Yaw-Bin Huang, Chen-Chun Kuo, Chung-Yu Chen

**Affiliations:** ^1^School of Pharmacy, Master Program in Clinical Pharmacy, Kaohsiung Medical University, Kaohsiung, Taiwan; ^2^Department of Pharmacy, Kaohsiung Medical University Hospital, Kaohsiung, Taiwan

## Abstract

Although previous studies demonstrated the risk of ischemic stroke (IS) in patients with head and neck cancer (HNC), the impact of oral antithrombotic therapy (OAT) on this risk has not yet been assessed. We aimed to evaluate the effectiveness and safety of OAT in patients with HNC treated with RT. This retrospective cohort study was performed using the National Health Insurance Research Database of Taiwan. A total of 37,638 patients diagnosed with HNC included in the study were classified as users and nonusers of OAT. Primary outcome was IS or transient ischemic attack (TIA), and secondary outcomes were death and major bleeding. The Cox proportional hazards model was used to calculate hazard ratios (HRs) and 95% confidence intervals (CIs). There was no significant difference in the risk of IS or TIA between patients on continuous OAT and nonusers (adjusted HR, 0.812; 95% CI, 0.199–3.309). The risk of major bleeding was not significantly different between the groups. From a national population database, we did not find an association between OAT and decreasing risk of ischemic stroke/TIA or increasing hazard of major bleeding.

## 1. Introduction

In Taiwan, head and neck cancer (HNC), which is diagnosed in more than 8,000 patients every year, is a leading cause of death [1, 2]. Clinical management of HNC includes surgery, radiation therapy (RT), and chemotherapy. Patients with early stage disease (stage I or II) are generally recommended single treatment modalities, such as surgery or RT, whereas treatment approaches utilizing combinations of several modalities are advised for patients with advanced stage disease (stage III or IV) [3].

RT is an important and effective treatment option in patients with HNC. However, radiation triggers an inflammatory response and precipitates damage to the vessel wall, eventually leading to the thickening of arterial walls, plaque formation, thrombosis, and altered blood flow or occlusion and stenosis of the artery. These disturbances in the vascular system are known to increase the risk of ischemic stroke and transient ischemic attack (TIA) [4]. Several studies have shown that RT in patients with HNC increased the risk of ischemic stroke and TIA [5–10].

Clinical trials that could robustly assess the efficacy of available treatment options in stroke prevention among patients with HNC treated with RT are limited, and the majority of the studies evaluated the efficacy of surgical management. Furthermore, no trial has thus far assessed pharmacological treatment options for primary or secondary stroke prevention. Oral antithrombotic therapy (OAT), including antiplatelet and anticoagulant agents, is frequently used in stroke prevention. However, the impact of OAT in patients with HNC treated with RT remains unclear [11].

The purpose of this study was to evaluate the efficacy and safety of OAT in patients with HNC treated with RT utilizing the National Health Insurance Research Database (NHIRD) of Taiwan and to provide a recommendation for general practitioners on the use of OAT in this patient population.

## 2. Methods

### 2.1. Data Source

This population-based retrospective cohort study used the 1997–2009 NHIRD registry for patients with catastrophic diseases, published by the National Health Research Institutes of Taiwan. Taiwan launched a single-payer National Health Insurance program on March 1, 1995; as of 2014, 99.9% of Taiwan's population was enrolled. Foreign nationals in Taiwan are also eligible for this program. The database contains records of patients with catastrophic illnesses, ambulatory care expenditures by visits, inpatient expenditures by admissions, details of ambulatory care orders, and details of inpatient orders.

This study was approved by the institutional review board of Kaohsiung Medical University Hospital (KMUH-IRB-EXEMPT-20150009). Current NHIRD and hospital regulations and guidelines did not mandate informed consent in this retrospective cohort study. All procedures performed were in accordance with the ethical standards of the institutional research committee and the directives of the Declaration of Helsinki.

### 2.2. Study Population

**Figure 1:**
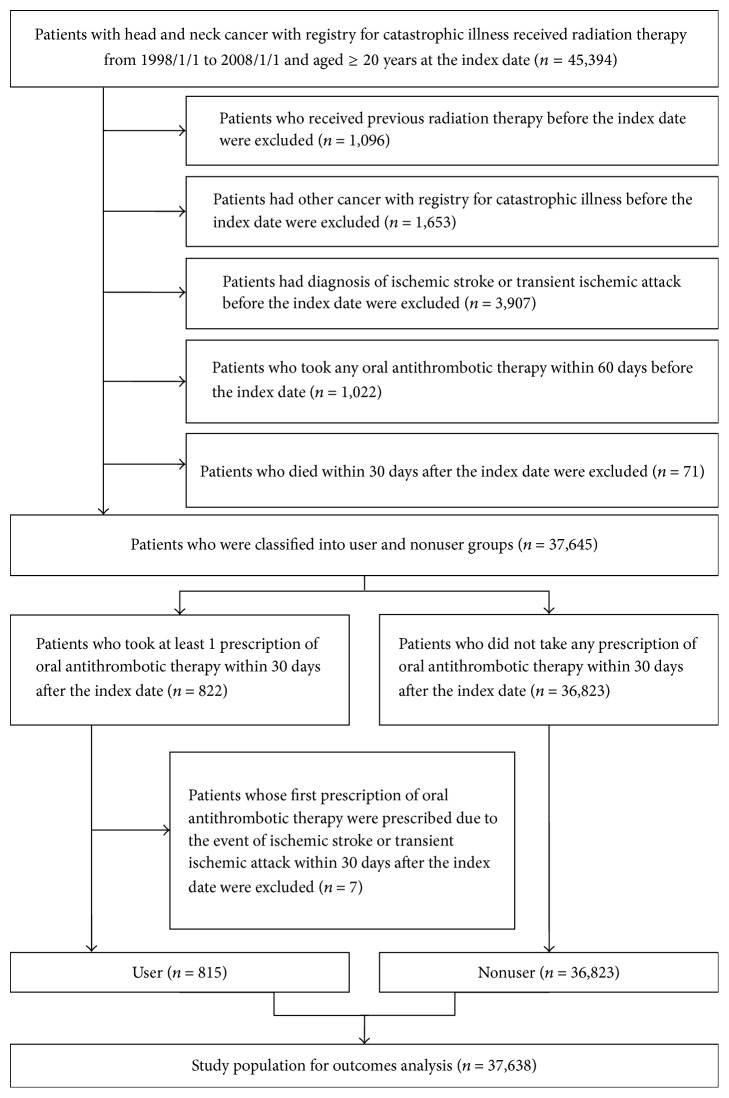
Study flowchart and results of study population selection.

The first date that a patient was treated with RT was defined as the index date. We included all patients aged ≥ 20 years at the index date, with a diagnosis of HNC (ICD-9-CM code: 140 to 149) registered as catastrophic illness and received RT between 1998/1/1 and 2008/1/1. We excluded patients (1) who received RT before the index date, (2) who had other cancer diagnoses (ICD-9-CM code: 150 to 239) registered as catastrophic illness before the index date, (3) who had a diagnosis of ischemic stroke or TIA (ICD-9-CM code: 433–438) before the index date, (4) who took any OAT within 60 days before the index date, (5) who died within 30 days after the index date, and (6) whose first OAT prescriptions were prescribed due to an episode of ischemic stroke or TIA within 30 days after the index date among OAT users. The flowchart of the study population selection is presented in Figure 1.

### 2.3. Exposure

Patients who had at least one prescription of any class of OAT within 30 days after the index date were categorized into the user group. Nonusers were patients who did not take any OAT prescriptions within 30 days after the index date. OAT included aspirin (daily dose, 81–100 mg), clopidogrel, ticlopidine, dipyridamole, cilostazol, and warfarin.

### 2.4. Outcomes

Primary outcome of this study was ischemic stroke or TIA. The following definitions were used for ischemic stroke and TIA: emergency department visit or hospitalization due to ischemic stroke or TIA (ICD-9-CM code: 433–438) with examination by computed tomography (CT) or magnetic resonance imaging (MRI), revascularization procedures, or admission to intensive care unit (ICU). Secondary outcomes were death from any cause and major bleeding. Major bleeding included admission to the emergency department and hospitalization due to intracranial hemorrhage and gastrointestinal bleeding. ICD-9-CM codes of major bleeding are presented in the supplementary information (eTable 1) in Supplementary Material available online at http://dx.doi.org/10.1155/2016/6205158.

### 2.5. Follow-Up

**Figure 2:**
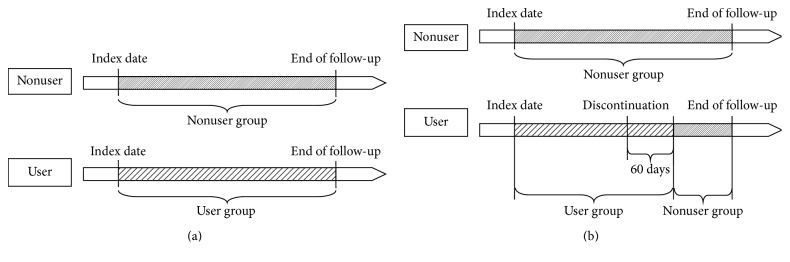
Two follow-up methods were performed: (a) follow-up method I and (b) follow-up method II.

Two follow-up methods were performed in this study. For follow-up method I, all patients were followed from the index date to the day of the occurrence of the event (Figure 2(a)). To confirm that patients in the user group were compliant with OAT without discontinuation, follow-up method II was performed. Specifically, OAT nonusers were followed from the index date to the day of the occurrence of the event. Patients in the user group were first followed as OAT users from the index date to 60 days after the date of OAT discontinuation. Discontinuation was defined as failure to fill the subsequent prescription during the 60-day period. OAT users who discontinued OAT were then included in the nonuser group 60 days after the date of discontinuation and were followed as OAT nonusers (Figure 2(b)). Censoring was applied for patients who did not reach the outcomes of interest at the end of follow-up and those in the nonuser group who started to take an oral antithrombotic agent.

### 2.6. Statistical Analysis

Descriptive statistics were performed to analyze baseline characteristics of the study population. Variables included age, gender, comorbidity (ICD-9-CM codes of comorbidity are presented in the supplementary information), and prescribed drugs. For continuous variables, data were presented as means and standard deviation (SD), and *t*-test was used to compare differences between users and nonusers. Categorical variables were presented as numbers and percentages, and the *χ* ^2^ test was used to compare differences between groups. The number of events, total follow-up person-years, and incidence of events (unit: events/1,000 person-years) were calculated. To assess the risk of events between users and nonusers, the Cox regression hazards model was used to calculate hazard ratios (HRs) and 95% confidence interval (CIs), and both univariate and multivariate analyses were performed. Covariates in multivariate analysis included age groups, gender, comorbidity, and prescribed drugs. In addition, 1-to-1 propensity score matching was performed to match users and nonusers [12]. Matched variables included age, gender, comorbidity, and prescribed drugs. For all statistical analyses performed in this study, *p* values < 0.05 were defined as significant. All data processing and statistical analyses were performed using SAS® software, version 9.4 (SAS Institute Inc., Cary, NC).

## 3. Results

In the present study, we identified 37,638 patients with HNC treated with RT within the defined time interval. Based on the definition of OAT exposure, there were 815 and 36,823 patients in the user and nonuser groups, respectively (Figure 1). The mean age of patients in the nonuser group (50.77 years) was significantly lower than that of patients in the user group (54.75 years, *p* < 0.001). Additionally, the ratio of male patients was significantly higher in the user group than in the nonuser group (nonuser, 85.37%; user, 87.98%; *p* = 0.0367). Furthermore, the rates of hypertension, diabetes mellitus, dyslipidemia, chronic kidney disease, heart failure, atrial fibrillation, ischemic heart disease, peripheral vascular disease, and deep venous thrombosis were significantly higher in the user group than in the nonuser group. Only the rate of gastrointestinal ulcer (nonuser, 8.06%; user, 8.59%; *p* = 0.5836) was similar between the two groups. Finally, the percentage of patients taking prescribed drugs (ACEIs, ARBs, beta blockers, CCBs, NSAIDs, PPIs, and statin) was significantly higher in the user group than in the nonuser group (Table 1 and eTable 2). The incidences of ischemic stroke, TIA, death from any cause, and major bleeding were higher in the user group than in the nonuser group, as determined by both follow-up methods (Table 2 and eTable 3).

The Cox proportional hazards model was used to assess the association of OAT with ischemic stroke and TIA. Univariate analysis using follow-up method I revealed that OAT users were at a significantly higher risk for ischemic stroke or TIA, compared with nonusers (crude HR, 1.923; 95% CI, 1.244–2.972; *p* = 0.0032). However, there was no significant difference between these two groups by multivariate analysis (adjusted HR, 1.214; 95% CI, 0.776–1.898; *p* = 0.3961). By follow-up method II, there were no significant differences between the groups by both univariate (crude HR, 1.395; 95% CI, 0.345–5.646; *p* = 0.6403) and multivariate analyses (adjusted HR, 0.812; 95% CI, 0.199–3.309; *p* = 0.7712; Table 3).

Multivariate analysis of ischemic stroke and TIA by follow-up method I also showed that older patients (age ≥ 45 years) were at a significantly higher risk of stroke and TIA than the reference population (age < 45 years). Additionally, the risk of stroke and TIA was significantly lower in females by multivariate analysis using follow-up method I. Among all comorbidity variables included in the multivariate analysis by follow-up method I, hypertension (adjusted HR, 1.483; *p* = 0.0002), diabetes mellitus (adjusted HR, 1.654; *p* < 0.0001), atrial fibrillation (adjusted HR, 2.467; *p* = 0.0026), and deep venous thrombosis (adjusted HR, 3.599; *p* = 0.0274) significantly increased the risk. Among the prescribed drugs evaluated in this study, ACEIs (adjusted HR, 1.697; *p* = 0.0350) and ARBs (adjusted HR, 1.983; *p* = 0.0305) were associated with significantly higher risk of ischemic stroke and TIA. Multivariate analysis by follow-up method II demonstrated similar results except for risk associated with ACEI and ARB use, which were not statistically significant (Table 4).

Univariate analysis by follow-up method I also revealed that OAT users were at a significantly higher risk of death from any cause than were nonusers (crude HR: 1.493; 95% CI: 1.085–2.053; *p* = 0.0138). In contrast, there was no significant difference in the risk of death between the two groups by multivariate analysis (adjusted HR, 1.306; 95% CI, 0.945–1.806; *p* = 0.1062). Using follow-up method II, both univariate and multivariate analyses determined that the risk of death was higher in OAT users than in nonusers (crude HR, 1.915; 95% CI, 1.228–2.988; *p* = 0.0042 versus adjusted HR, 1.662; 95% CI, 1.059–2.607; *p* = 0.0271). However, univariate and multivariate analyses by follow-up methods I and II revealed that the risk of major bleeding was not significantly different between users and nonusers (Table 3).

The total number of patients in the nonuser group was 36,823 in the present study. After 1-to-1 propensity score matching, there were 815 patients each in the nonuser and user groups. As shown in Table 1, all baseline characteristics, age, gender, comorbidity, and prescribed drugs were well-matched and evenly distributed between the user and nonuser groups, and no significant differences in any of the variables were observed between users and nonusers (Table 1).

The incidences of ischemic stroke, TIA, and death were higher in users than in nonusers as determined by follow-up method I; in contrast, the incidence of major bleeding was lower in the user group than in the nonuser group. Conversely, analysis performed by follow-up method II showed that while the incidences of ischemic stroke and TIA were higher in users than in nonuser group, the incidence of death and major bleeding was higher in users than in nonusers (Table 2). Using the Cox proportional hazards model with follow-up method I, both univariate (crude HR, 1.727; 95% CI, 0.849–3.512; *p* = 0.1313) and multivariate analyses (adjusted HR, 1.609; 95% CI, 0.779–3.325; *p* = 0.1988) showed no significantly increased risk of ischemic stroke and TIA. This risk was not significantly different when the same analysis was performed by follow-up method II. Finally, no significant differences in the risk for death from any cause or major bleeding were found between users and nonusers by univariate or multivariate analyses using the Cox proportional hazards model and follow-up methods I and II (Table 3).

## 4. Discussion

In this population-based cohort study to examine the efficacy and safety of OAT in patients with HNC treated with RT, we found that OAT did not significantly reduce the risk of the primary outcome of this study, that is, ischemic stroke and TIA. Among the secondary outcomes evaluated in the present study, OAT use was associated with increased mortality, although it did not significantly increase the risk of major bleeding.

The mean age of patients included in the present study ranged from 50 to 54 years, which was similar to that reported for patients with HNC by the annual Taiwan cancer registry report in 2012 (50–60 years) [2]. Additionally, the distribution of patient age in our study was similar to that reported by Huang et al. [7]; the highest percentage of patients was in the 45–54 age group. The percentage of male patients in our study (nonusers, 85%; users, 88%), albeit slightly lower than that observed in HNC patients in Taiwan cancer registry annual report (approximately 90%) [2], was similar to that observed in a study by Chu et al. (85%) [5]. Furthermore, while the ratio of nonuser patients with hypertension (nonusers, 15.27%) was similar to that reported by Chu and colleagues (16.1%), their cohort included more diabetic patients (13.1%) than the present study (nonusers, 9.23%) [5]. However, baseline characteristics of patients in the study by Huang et al. showed that the percentages of patients with hypertension (5.2%), diabetes (5.2%), and hyperlipidemia (0.4%) were distinctively lower than those in our study [7]. These differences were likely due to the differences in databases and definitions of comorbidities between the two studies. In the present study, the mean age of users was higher than nonusers, and the percentage of patients older than 65 years of age was higher in the user group. Furthermore, more OAT users suffered from cardiovascular diseases and were prescribed related drugs.

In the present study, the incidence rate of ischemic stroke and TIA was 5.57/1,000 person-years in the nonuser group and 10.32/1,000 person-years in the nonuser group by analysis using follow-up method I, which was lower than that reported by Chu et al. (between 11.5 and 11.8/1,000 person-years) [5]. One potential reason for this difference is the strict definition used for events in our study. Specifically, episodes of ischemic stroke or TIA were not only determined by the diagnostic ICD-9-CM codes but also determined by the codes for relevant imaging studies, revascularization procedures, and ICU admission. This approach enabled the precise detection of events. The Cox proportional hazards model applied with two follow-up methods and adjustment for all variables did not show any statistically significant differences between users and nonusers. Using this approach, stroke-related risk factors as variables such as age, gender, hypertension, diabetes mellitus, atrial fibrillation, and deep venous thrombosis showed a significantly increased risk of ischemic stroke and TIA [13]. Other variables, namely, ACEI and ARB use, were associated with a significantly increased risk of ischemic stroke and TIA, which might reflect the number of patients treated with ACEI or ARB for hypertension [14]. We also performed 1-to-1 propensity score matching to correct the imbalance in baseline characteristics between users and nonusers and no significantly difference was found by analysis with follow-up methods I and II.

No studies to date have evaluated the efficacy of OAT for primary prevention of ischemic stroke or TIA in patients with HNC undergoing RT [11]. Our findings demonstrated that OAT did not significantly reduce the risk of stroke and TIA in patients with HNC treated with RT, which might be due to antiplatelet resistance and low levels of international normalized ratio (INR) in patients taking oral vitamin K antagonists. Extensive focal inflammation and necrosis of vasa vasorum and adventitium were previously shown in patients treated with RT [11]. Chronic inflammation can cause antiplatelet resistance with accelerated platelet turnover [15]. In addition, an* in vivo* study revealed that low-dose aspirin did not have a significant effect on inhibition of platelet aggregation in irradiated mice [16]. Warfarin, an oral vitamin K antagonist, is usually used for stroke prevention in patients with atrial fibrillation and mechanical valve replacement. Guidelines suggest that INR levels should be between 2.0 and 3.0 for atrial fibrillation patients and between 2.5 and 3.5 for patients with mechanical valve replacement [13, 17]. Despite the lack of population-based studies on INR levels in Taiwanese patients, two hospital-based studies [18, 19] in Taiwan indicated that most patients on warfarin had INR levels of less than 2.0 (71.2% and 70% by Kuo et al. [18] and Yu et al. [19], resp.), which were below the recommended values.

To our knowledge, this is the first study to evaluate the efficacy of OAT in patients with HNC treated with RT. This cohort study utilized a large population size and longitudinal claims data. Additionally, our database included registry data for patients with catastrophic illness that allowed for precise identification of HNC cases. However, several limitations in our study need to be addressed. First, our study database did not contain information related to personal lifestyle choices such as smoking and alcohol consumption that are known risk factors for ischemic stroke [13]. Second, detailed information on physical examination, laboratory tests, imaging studies, clinical cancer stage, and TOAST etiologies of the stroke patients were not available in the database. For example, body weight, blood pressure, and low-density lipoprotein cholesterol levels are also associated with risk of ischemic stroke [13]. Imaging studies also provide additional information on the severity of carotid artery stenosis, and cancer staging can inform on mortality rates. Third, our database did not provide information on the doses used in each RT session. A study by Haynes et al. failed to demonstrate an effect of radiation dose on the risk of stroke in patients with HNC; radiation doses in stroke patients evaluated in that study ranged from 59.4 to 76.8 Gy [20]. Thus, although we could not include radiation dose as a variable in the regression models, its potential effect on the risk of ischemic stroke might be minimal. Fourth, not all ICD-9-CM codes included in our study outcomes were validated in the NHIRD. For ischemic stroke or TIA, the accuracy of the NHIRD in recording ischemic stroke diagnoses with the ICD-9-CM codes of 433.xx and 434.xx was high (94%) [21]. In contrast, we used the ICD-9-CM codes of 433.xx to 438.xx in the present study. While 435.xx to 438.xx were not yet validated, we confirmed the ischemic stroke and TIA events not only by these diagnosis codes but also by the procedure codes for imaging studies, revascularization procedures, and ICU admission. Fifth, we did not add each kind of statin use into our Cox regression models due to small sample size. The confidence intervals of each kind of statin were too wide to be presented.

## 5. Conclusions

In conclusion, OAT did not significantly reduce the risk of ischemic stroke or TIA in patients with HNC treated with RT. Furthermore, no significant major bleeding was observed in patients taking OAT. Furthermore, randomized clinical trials are needed to comprehensively evaluate the efficacy and safety of OAT in this study population.

## Supplementary Material

The supplementary material included three tables. Etable 1 was ICD-9-CM codes used in this study. Etable 2 was utilization of statin in study population. Etable 3 was incidence of ischemic stroke and TIA in study population, and divived into method I and method II.

## Figures and Tables

**Table 1:** Baseline characteristics of head and neck cancer patients with radiation therapy and study population after propensity score matching.

Variables	Study population					Study population after PSM				
	Nonuser (*n* = 36,823)		User (*n* = 815)		*p* value	Nonuser (*n* = 815)		User (*n* = 815)		*p* value
	*n*	%	*n*	%		*n*	%	*n*	%	
*Age (years)*										
<45	11561	31.40	172	21.10	<0.0001	173	21.23	172	21.10	0.9989
45 to 54	12835	34.86	259	31.78		257	31.53	259	31.78	
55 to 64	7341	19.94	208	25.52		210	25.77	208	25.52	
65 to 74	3789	10.29	126	15.46		123	15.09	126	15.46	
≥75	1297	3.52	50	6.14		52	6.38	50	6.14	
Mean ± SD	50.77	±11.79	54.75	±11.84	<0.0001	54.51	±12.12	54.75	±11.84	0.6823
*Gender*										
Men	31434	85.37	717	87.98	0.0367	722	88.59	717	87.98	0.7002
Women	5389	14.63	98	12.02		93	11.41	98	12.02	
*Comorbidity*										
Hypertension	5623	15.27	248	30.43	<0.0001	257	31.53	248	30.43	0.6298
Diabetes mellitus	3399	9.23	178	21.84	<0.0001	183	22.45	178	21.84	0.7655
Dyslipidemia	1828	4.96	72	8.83	<0.0001	64	7.85	72	8.83	0.4737
Chronic kidney disease	279	0.76	23	2.82	<0.0001	23	2.82	23	2.82	1.0000
Heart failure	314	0.85	38	4.66	<0.0001	34	4.17	38	4.66	0.6297
Atrial fibrillation	162	0.44	16	1.96	<0.0001	13	1.60	16	1.96	0.5740
Gastrointestinal ulcer	2968	8.06	70	8.59	0.5836	76	9.33	70	8.59	0.6028
Ischemic heart disease	1233	3.35	139	17.06	<0.0001	137	16.81	139	17.06	0.8949
Peripheral vascular disease	87	0.24	8	0.98	<0.0001	6	0.74	8	0.98	0.5914
Deep venous thrombosis	41	0.11	11	1.35	<0.0001	9	1.10	11	1.35	0.6527
*Prescribed drug*										
ACEI	550	1.49	55	6.75	<0.0001	51	6.26	55	6.75	0.6878
ARB	274	0.74	30	3.68	<0.0001	37	4.54	30	3.68	0.3825
Beta blocker	118	0.32	8	0.98	0.0012	8	0.98	8	0.98	1.0000
CCB	51	0.14	13	1.60	<0.0001	15	1.84	13	1.60	0.7030
NSAIDs	13898	37.74	402	49.33	<0.0001	415	50.92	402	49.33	0.5196
PPI	1335	3.63	82	10.06	<0.0001	83	10.18	82	10.06	0.9346
Statin	84	0.23	13	1.60	<0.0001	10	1.23	13	1.60	0.5287

PSM, propensity score matching; ACEI, angiotensin-converting-enzyme inhibitor; ARB, angiotensin II receptor blocker; CCB, calcium channel blocker; NSAIDs, nonsteroidal anti-inflammatory drugs; PPI, proton pump inhibitor.

**Table 2:** Incidence of study outcomes in head and neck cancer patients with radiation therapy between oral antithrombotic therapy users and nonusers.

Follow-up method	Outcome	Nonuser			User		
		Number of events	Total person-years	Incidence rate^†^	Number of events	Total person-years	Incidence rate^†^
Study population: nonuser (*n* = 36,823); user (*n* = 815)							
Method I	Ischemic stroke or TIA	600	107648.01	5.57	21	2034.16	10.32
	Death	1220	108714.85	11.22	39	2087.92	18.68
	Major bleeding	1730	106366.72	16.26	43	2037.69	21.10
Method II	Ischemic stroke or TIA	613	109023.26	5.62	2	318.45	6.28
	Death	1237	110096.69	11.24	20	318.62	62.77
	Major bleeding	1762	107720.68	16.36	14	314.46	44.52

Study population after 1-to-1 propensity score matching: nonuser (*n* = 815); user (*n* = 815)							
Method I	Ischemic stroke or TIA	12	1995.20	6.01	21	2034.16	10.32
	Death	26	2007.16	12.95	39	2087.92	18.68
	Major bleeding	46	1928.04	23.86	43	2037.69	21.10
Method II	Ischemic stroke or TIA	25	3370.45	7.42	2	318.45	6.28
	Death	43	3389.00	12.69	20	318.62	62.77
	Major bleeding	78	3281.99	23.77	14	314.46	44.52

TIA, transient ischemic attack.

^†^Unit of incidence rate: 1,000 person-years.

**Table 3:** Association of study outcomes in head and neck cancer patients with radiation therapy between oral antithrombotic therapy users and nonusers.

Follow-up method	Outcome	Crude HR	95% CI		*p* value	Adjusted HR^†^	95% CI		*p* value
			Lower	Upper			Lower	Upper	
Study population									
Method I	Ischemic stroke or TIA	1.923	1.244	2.972	0.0032	1.214	0.776	1.898	0.3961
	Death	1.493	1.085	2.053	0.0138	1.306	0.945	1.806	0.1062
	Major bleeding	1.237	0.914	1.675	0.1678	0.951	0.699	1.294	0.7490
Method II	Ischemic stroke or TIA	1.395	0.345	5.646	0.6403	0.812	0.199	3.309	0.7712
	Death	1.915	1.228	2.988	0.0042	1.662	1.059	2.607	0.0271
	Major bleeding	1.391	0.819	2.363	0.2215	1.057	0.620	1.802	0.8397

Study population after 1-to-1 propensity score matching									
Method I	Ischemic stroke or TIA	1.727	0.849	3.512	0.1313	1.609	0.779	3.325	0.1988
	Death	1.442	0.878	2.369	0.1484	1.511	0.916	2.493	0.1061
	Major bleeding	0.888	0.586	1.346	0.5758	0.875	0.575	1.330	0.5313
Method II	Ischemic stroke or TIA	0.666	0.142	3.130	0.6068	0.506	0.110	2.330	0.3816
	Death	1.458	0.833	2.550	0.1865	1.450	0.822	2.556	0.1995
	Major bleeding	0.951	0.512	1.768	0.8748	0.853	0.460	1.583	0.6149

HR, hazard ratio; CI, confidence interval; TIA, transient ischemic attack.

^†^Adjusted variables included age, gender, comorbidity, and prescribed drugs.

**Table 4:** Cox proportional hazard model of ischemic stroke and transient ischemic attack in head and neck cancer patients with radiation therapy between oral antithrombotic therapy users and nonusers.

Variables	Follow-up method I				Follow-up method II			
	Adjusted HR^†^	95% CI		*p* value	Adjusted HR^†^	95% CI		*p* value
		Lower	Upper			Lower	Upper	
*User versus nonuser*	1.214	0.776	1.898	0.3961	0.812	0.199	3.309	0.7712
*Age (years)*								
<45	1.000	—	—	—	1.000	—	—	—
45 to 54	2.509	1.954	3.221	<0.0001	2.503	1.949	3.214	<0.0001
55 to 64	3.561	2.742	4.625	<0.0001	3.567	2.746	4.634	<0.0001
65 to 74	4.498	3.357	6.028	<0.0001	4.403	3.279	5.912	<0.0001
≥75	5.722	3.794	8.631	<0.0001	5.615	3.707	8.505	<0.0001
*Gender*								
Men	1.000	—	—	—	1.000	—	—	—
Women	0.708	0.567	0.884	0.0023	0.708	0.567	0.885	0.0024
*Comorbidity*								
Hypertension	1.483	1.209	1.819	0.0002	1.492	1.215	1.832	0.0001
Diabetes mellitus	1.654	1.310	2.088	<0.0001	1.652	1.305	2.091	<0.0001
Dyslipidemia	0.821	0.588	1.145	0.2445	0.838	0.600	1.170	0.3000
Chronic kidney disease	1.146	0.508	2.583	0.7427	1.188	0.526	2.679	0.6788
Heart failure	0.993	0.485	2.032	0.9847	1.057	0.517	2.162	0.8799
Atrial fibrillation	2.467	1.369	4.444	0.0026	2.521	1.398	4.547	0.0021
Gastrointestinal ulcer	0.920	0.691	1.224	0.5659	0.926	0.696	1.233	0.5993
Ischemic heart disease	1.213	0.873	1.686	0.2500	1.224	0.877	1.710	0.2352
Peripheral vascular disease	0.924	0.229	3.723	0.9118	0.956	0.237	3.852	0.9496
Deep vein thrombosis	3.599	1.154	11.225	0.0274	3.761	1.206	11.724	0.0224
*Prescribed drugs*								
ACEI	1.697	1.038	2.775	0.0350	1.549	0.922	2.602	0.0981
ARB	1.983	1.066	3.688	0.0305	1.874	0.982	3.578	0.0568
Beta blocker	1.592	0.588	4.313	0.3603	1.631	0.602	4.420	0.3360
CCB	0.000	0.000	3.62 × 10^144^	0.9530	0.000	0.000	2.63 × 10^160^	0.9580
NSAIDs	0.929	0.781	1.105	0.4043	0.939	0.789	1.117	0.4773
PPI	0.772	0.431	1.381	0.3833	0.797	0.445	1.426	0.4447
Statin	1.225	0.296	5.066	0.7791	1.438	0.349	5.935	0.6153

HR, hazard ratio; CI, confidence interval; ACEI, angiotensin-converting-enzyme inhibitor; ARB, angiotensin II receptor blocker; CCB, calcium channel blocker; NSAIDs, nonsteroidal anti-inflammatory drugs; PPI, proton pump inhibitor.

^†^Adjusted variables included age, gender, comorbidity, and prescribed drugs.
