# Supplementary material for: Evaluating the Primary Prevention of Ischemic Stroke of Oral Antithrombotic Therapy in Head and Neck Cancer Patients with Radiation Therapy
Source: Biomed Res Int. 2016 Nov 21;2016:6205158. doi: 10.1155/2016/6205158 (PMC5136628; doi:10.1155/2016/6205158)
Supplement: Supplementary file 1 — The supplementary material included three tables. Etable 1 was ICD-9-CM codes used in this study. Etable 2 was utilization of statin in study population. Etable 3 was incidence of ischemic stroke and TIA in study population, and divived into method I and method II. [file 6205158.f1.pdf]

Supplementary information

eTable 1. ICD-9-CM codes in this study

Disease	ICD-9				
Head and neck cancer	140	141	142	143	144
	145	146	147	148	149
Other cancer	150 - 239				
Ischemic stroke/transient ischemic attack	433	434	435	436	437
	438				
Hypertension	401	402	403	404	405
Diabetes mellitus	250				
Dyslipidemia	272				
Chronic kidney disease	585				
Heart failure	428				
Atrial fibrillation	427.31				
Gastrointestinal ulcer	531	532	533	534	
Ischemic heart disease	410	411	412	413	414
Peripherial vascular disease	443				
Major bleeding	430	431	432	531.0	531.2
	531.4	531.6	532.0	532.2	532.4
	532.6	533.0	533.2	533.4	533.6
	534.0	534.2	534.4	534.6	535.01
	535.11	535.21	535.31	535.41	535.51
	535.61	537.83	562.02	562.03	562.12
	562.13	56881	562.12	569.3	569.85
	578				

eTable 2. Utilization of statin in study population

Variables	Study population					Study population after PSM				
	Non-user (n = 36,823)		User (n = 815)			Non-user (n = 815)		User (n = 815)		
	n	%	n	%	p-value	n	%	n	%	p-value
**Prescribed drug**										
Statin	84	0.23	13	1.60	<.0001	10	1.23	13	1.60	0.5287
Atorvastatin	38	0.10	8	0.98	<.0001	7	0.86	8	0.98	0.7953
Fluvastatin	7	0.02	1	0.12	0.0446	1	0.12	1	0.12	1.0000
Lovastatin	4	0.01	1	0.12	0.0061	0	0.00	1	0.12	0.3172
Simvastatin	9	0.02	1	0.12	0.0887	0	0.00	1	0.12	0.3172
Pravastatin	11	0.03	1	0.12	0.1420	2	0.25	1	0.12	0.5633
Rosuvastatin	10	0.03	1	0.12	0.1145	0	0.00	1	0.12	0.3172
≥ 2 kinds of statin	5	0.01	0	0.00	0.7394	51	6.26	55	6.75	0.6878

eTable 3. The incidence of ischemic stroke and TIA in study population, stratified by method I and method II.

		Non-user			User		
**Follow-up method**	**Outcome**	**No. of events**	**Total person-years**	**Incidence**	**No. of events**	**Total person-years**	**Incidence**†
**Study population: non-user (n = 36,823); user (n = 815)**							
Method I	Ischemic stroke or TIA	600	107648.01	5.57	21	2034.16	10.32
	Ischemic stroke	559	107648.01	5.19	19	2034.16	9.34
	TIA	26	107648.01	0.24	2	2034.16	0.98
	Ischemic stroke and TIA	15	107648.01	0.14	0	2034.16	0.00
Method II	Ischemic stroke or TIA	613	109023.26	5.62	2	318.45	6.28
	Ischemic stroke	570	109023.26	5.23	2	318.45	6.28
	TIA	28	109023.26	0.26	0	318.45	0.00
	Ischemic stroke and TIA	15	109023.26	0.14	0	318.45	0.00
**Study population after 1-to-1 propensity score matching: non-user (n = 815); user (n = 815)**							
Method I	Ischemic stroke or TIA	12	1995.20	6.01	21	2034.16	10.32
	Ischemic stroke	11	1995.20	5.51	19	2034.16	9.34
	TIA	1	1995.20	0.50	2	2034.16	0.98
	Ischemic stroke and TIA	0	1995.20	0.00	0	2034.16	0.00
Method II	Ischemic stroke or TIA	25	3370.45	7.42	2	318.45	6.28
	Ischemic stroke	22	3370.45	6.53	2	318.45	6.28
	TIA	3	3370.45	0.89	0	318.45	0.00
	Ischemic stroke and TIA	0	3370.45	0.00	0	318.45	0.00
